# First Analysis of Human *Coccidioides* Isolates from New Mexico and the Southwest Four Corners Region: Implications for the Distributions of *C. posadasii* and *C. immitis* and Human Groups at Risk

**DOI:** 10.3390/jof5030074

**Published:** 2019-08-10

**Authors:** Paris S. Hamm, Miriam I. Hutchison, Pascale Leonard, Sandra Melman, Donald O. Natvig

**Affiliations:** 1 Department of Biology, University of New Mexico, Albuquerque, NM 87131, USA; 2 New Mexico Department of Health, Scientific Laboratory Division, 1101 Camino de Salud NE, Albuquerque, NM 87102, USA; 3 New Mexico Department of Health, Epidemiology and Response Division, Santa Fe, NM 87505, USA

**Keywords:** *Coccidioides*, coccidioidomycosis, New Mexico, American Indian, Valley Fever

## Abstract

Coccidioidomycosis (Valley Fever) is a disease caused by species of *Coccidioides*. The disease is endemic to arid regions of the Southwestern US and while most common in CA and AZ is also present in NM. We present the first genetic analysis of clinical isolates from NM. Travel and demographic information was available for a number of patients, which included individuals from NM and the Southwestern US Four Corners region. Multi-gene phylogenetic analyses revealed the presence of both *C. posadasii* and *C. immitis*. While NM is predicted to be within the endemic range for *C. posadasii*, our results expand the known range of *C. immitis*, often considered to be the “California species”. Five of eight infections for which patient ethnicity existed occurred in Native Americans, and two occurred in African Americans. Several isolates came from the northwestern part of NM—outside the predicted “highly-endemic” region. Our study suggests Native Americans represent an unrecognized at-risk group, and it provides a foundation for better defining the geographic distribution of the *Coccidioides* species and for preventing exposure among populations at risk. In the course of this study, we developed a reliable PCR-based method to distinguish species targeting regions of the mitochondrial genome.

## 1. Introduction

Coccidioidomycosis, commonly known as Valley Fever, is caused by the soil-dwelling fungi *Coccidioides immitis* and *C. posadasii*. As is common for fungi that infect humans and animals, species of *Coccidioides* are dimorphic in terms of their life cycle, growing saprobically as multicellular filaments on non-living organic matter and, upon entry into a host lung, growing pathogenically in a yeast-like spherule stage [1]. The endospores formed by spherules can disseminate within a host but are not transmissible to new hosts. Outside a living host, species of *Coccidioides* form arthroconidia (asexual spores) that can become airborne via soil disturbance and can be inhaled by potential hosts [2]. Although coccidioidomycosis presents in about 40% of patients as a pulmonary infection, a chronic and disseminated form of coccidioidomycosis, often resulting in lifelong treatment, occurs in roughly 5% of patients [3,4].

Although arthroconidia can be detected in soil and in dust, the specific niches of *Coccidioides* species are unknown [5]. The disease has been characterized as endemic to arid environments that include the Southwestern United States, Central and South America and Mexico [6]. In the United States, highly endemic hotspots have been documented in California and Arizona [7,8,9]. Although less prevalent, Valley Fever is present in Nevada, Colorado, Texas, New Mexico, Utah and Washington state [10,11,12,13].

Molecular and phenotypic analyses have resulted in *Coccidioides* being divided into two species: *C. immitis*, known primarily from California, and *C. posadasii*, recognized originally as the non–California group [6]. While clinical diagnosis and treatment of coccidioidomycosis have not depended on identifying isolates to species, medical and scientific communities are becoming more cognizant of the potential need to recognize previously undetected phenotypic, morphological, ecological, and genetic differences between species. Multi-locus genetic analyses and whole-genome studies have attempted to map the distribution of these species. To date, however, the isolates studied have been mainly limited to California, Arizona, Central America, Mexico, and Texas [14,15], and the genetics of isolates from New Mexico have not been examined. The study reported here employed genetic analyses of 18 isolates collected from patients diagnosed with coccidioidomycosis from diverse locations across New Mexico and the Four Corners region. Although Southern New Mexico has been recognized as part of the endemic region for *Coccidioides* [12,16], several of the isolates examined here were from Northern and Central New Mexico, suggesting a broad range for *Coccidioides* that includes the Four Corners region. Also noteworthy is the fact that isolates of both *C. immitis* and *C. posadasii* were obtained from patients in New Mexico with *C. immitis* being obtained from a patient who resides in San Juan County, NM.

The risk for coccidioidomycosis has been reported as much higher among some ethnic groups, particularly African Americans and Filipinos [17]. In these ethnic groups, the risk for disseminated coccidioidomycosis is ten-fold that of the general population [18]. Health records from patients in our study suggest the possibility that Native American Indians represent an additional risk group for disseminated disease.

## 2. Materials and Methods 

### 2.1. Sample Collection and Patient Information

Eighteen human clinical specimens from seventeen patients diagnosed with coccidioidomycosis obtained between 2013 and 2017 were submitted to the New Mexico Department of Health (NMDOH) Scientific Laboratory Division (SLD). Coccidioidomycosis is a reportable condition in New Mexico. The NMDOH collects information on all confirmed and probable cases (case status definitions follow CSTE standards) who reside in New Mexico at the time of diagnosis to look for potential risk factors via chart review and data extraction. When possible, patient information was noted with specific interest in type of infection, residency, travel history, occupation, race/ethnicity, gender, age, and medical history (Table 1). 

### 2.2. Molecular Methods

DNA was extracted at the NMDOH SLD from *Coccidioides* isolates using the PrepMan^®^ Ultra Reagent method (Applied Biosystems, Foster City, CA) and stored at −80 °C until further processing. The DNA extractions were transferred to the Natvig Laboratory at the University of New Mexico where a 1/10 DNA dilution of each preparation was used for PCR amplification of three AFToL (Assembling the Fungal Tree of Life)-designated nuclear gene regions in addition to a serine proteinase gene region diagnostic for species (Table 2). For the serine proteinase, MCM7, and RPB1 genes, primers were designed to amplify and sequence regions that we determined from comparisons of sequences in GenBank to be diagnostic in distinguishing between the two *Coccidioides* species. Serine proteinase, specifically, was chosen as a target based on results presented by Koufopanou et al. [19]. ITS amplification and sequencing involved the entire ITS1-5.8S rRNA-ITS2 region. 

We designed a primer pair, with one primer anchored in a sequence unique to *C. posadasii*, to amplify a mitochondrial intron sequence from *C. posadasii* but not *C. immitis*. A second primer set was designed to amplify a portion of the first cox1 exon along with an upstream intergenic region in both *Coccidioides* species but with the potential to differentiate *Coccidioides* from other Onygenales (Table 2). 

All PCR reactions began with an initial step at 95 °C for 5 min. This was followed by 35 cycles of 94 °C for 30 s, a gene-specific annealing temperature (Table 2) for 30 s, then 72 °C for 45 s with a final extension at 72 °C for 7 min. PCR products were cleaned with ExoSAP-IT (Thermo Fisher Scientific, Waltham, MA, USA) before DNA sequencing using BigDye v3.1 (Applied Biosystems, Foster City, CA, USA) chain termination with the Big Dye STeP protocol [20]. Forward and reverse sequences were assembled and edited with Sequencher 5.1 (Gene Codes, Ann Arbor, MI, USA). Sequences were deposited in GenBank under accessions MH748760–MH748777 for serine proteinase; MH748742–MH748759 for MCM7; MH748724–MH748741 for RPB1; and MH725244–MH725261 for ITS (Table 3).

### 2.3. Phylogenetic Analysis

Sequences for the four gene regions were aligned individually using Clustal Omega version 1.2.45 [21]. For outgroup sequences, each alignment included the appropriate homologous gene region from *Aspergillus steynii* from the GenBank accessions (Table 3). This species was chosen as an outgroup because it had a clear homolog for each of the four gene regions examined. The serine proteinase in particular, appears to undergo rapid evolution and perhaps gene loss, and as a result, it was difficult to identify clear orthologs in many other species of Eurotiomycetes.

Tree-building analyses employed maximum likelihood analysis with PHYLIP (version 3.695) DNAMLK and parsimony analysis with PHYLIP DNAPARS [22]. In each case, tree building employed 1000 bootstrap datasets. Analyses were done on each of the four gene alignments separately, as well as on a concatenated alignment of all four genes. The concatenated alignment has been deposited at TreeBase (Submission ID 24737). 

### 2.4. Ethics Statement

All patient data analyzed for this study were anonymized.

## 3. Results

**Figure 1:**
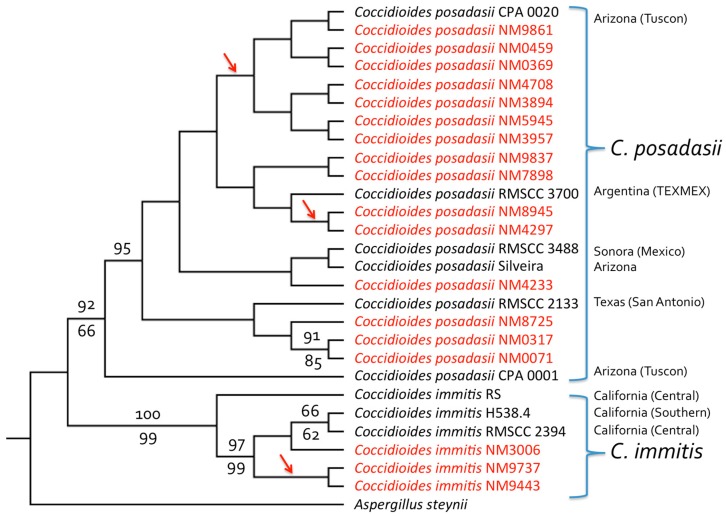
Four-gene phylogeny (DNAMLK) for clinical *Coccidioides* isolates from New Mexico and the Four Corners region. The tree was derived from a concatenated alignment of partial sequences from four gene regions: Serine protease, MCM7, RPB1, and ITS; trees for individual genes are shown in Figure S1 (with all four genes agreeing in terms of species separation). Both *C. posadasii* and *C. immitis* isolates were present among those obtained from patients in New Mexico (shown in red). As expected, based on previous analyses of isolates from California, Arizona and Texas, most NM isolates were from *C. posadasii* (*C. immitis* being known primarily from CA). One patient infected with *C. immitis* (isolates NM9443 and NM9737) was a resident of the Four Corners region of Utah, while another (isolate NM3006) was from the Four Corners region of NM with no apparent travel history to California. GenBank accession numbers are given in Table 3. Bootstrap values (percentage of 1000 replicates) greater than 60% are shown for maximum likelihood analysis above the branches and for parsimony analysis below the branches. The tree was rooted with *Aspergillus steynii*. Maximum likelihood and parsimony analyses performed without sequences from *A. steynii*, and employing midpoint rooting, separated *C. immitis* and *C. posadasii* clades with 100% bootstrap support and placed the root between the two species (results not shown). Arrows indicate branches leading to isolates with identical sequences.

All four gene regions examined were capable of distinguishing between the two *Coccidioides* species, based on comparisons with sequences reported in Genbank. Three of the isolates were revealed to be *C. immitis*, while the remaining 15 were *C. posadasii*. Isolates NM3006, NM9443, and NM9737 were clustered together as a *C. immitis* clade in single-locus trees and in the concatenated four-gene phylogeny (Figure 1 and Figure S1). Both NM9443 and NM9737 came from a single patient in Utah whose type of infection was unknown (Table 1). Isolate NM3006 was from a 60-year-old male from San Juan County, New Mexico, with disco-vertebral osteomyelitis due to *C. immitis*. Human coccidioidomycosis due to *C. posadasii* was represented by twelve cases of pulmonary infections (one also had a facial lesion), one of osteomyelitis, and one knee infection. The type of infection associated with the remaining patient was unknown (Table 1). Five of eight infections for which patient ethnicity was known occurred in Native Americans, while two of the eight occurred in African Americans (Table 1).

**Figure 2:**
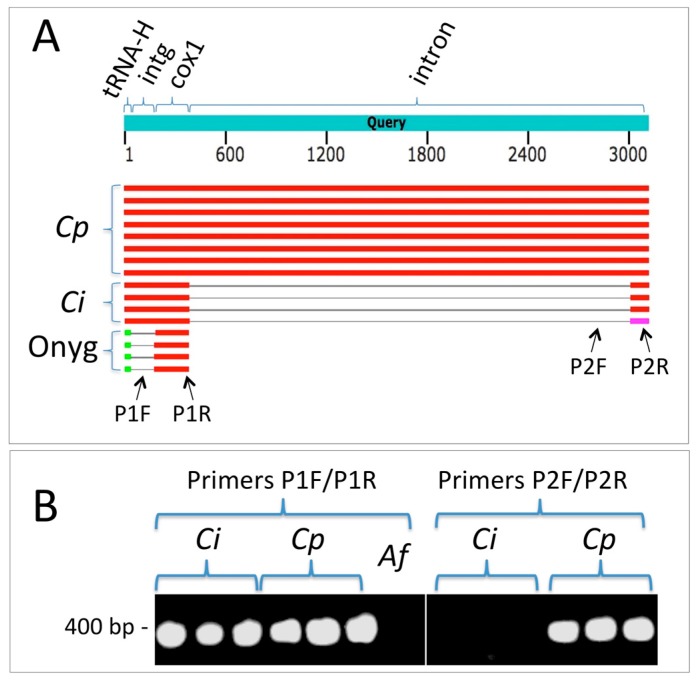
An mtDNA region can be employed to distinguish between *C. immitis* and *C. posadasii* using PCR. (A) Summary results from a BLASTN search employing the cox1 mtDNA region from *C. posadasii* strain C735 delta SOWgp (GenBank: ACFW01000039). *Cp* = hits against *C. posadasii* sequences, *Ci* = hits against *C. immitis*, and Onyg = hits against other Onygenales (*Microsporum canis* CBS 113480, *Trichophyton interdigitale* M8436, *Trichophyton mentagrophytes* TIMM 2789, and *Trichophyton interdigitale* H6). Note that the gap in the intron of *C. immitis* relative to *C. posadasii* represents a large indel. The intergenic region (intg) between the histidine tRNA and cox1 is conserved between the two *Coccidioides* species but not between the latter and other Onygenales. The sequence corresponding to primer P1F is present in both *Coccidioides* species but not in other genera for which sequences are currently available in Genbank. The P1R sequence is contained within the first cox1 exon and is conserved in both species. The P2F primer sequence is entirely absent from *C. immitis*. The P2R primer sequence is wholly contained within the *C. posadasii* intron but spans the predicted exon–intron border of *C. immitis*. (B) Primer pair P1F/P1R amplifies a fragment from both *C. immitis* and *C. posadasii* (*Aspergillus fumigatus*, *Af*, included as negative control). Primer pair P2F/P2R amplifies a fragment from *C. posadasii* only (although this fragment was amplified in all 15 *C. posadasii* strains, results are shown for only three strains.

In addition to the four nuclear genes examined, we explored the possibility that mitochondrial DNA (mtDNA) regions might be useful in distinguishing between *C. immitis* and *C. posadasii*. In comparisons of the sequences available in GenBank, it appeared that the first intron of the cytochrome c oxidase I (cox1) gene possesses a large insertion/deletion that separates the two species. This allowed the design of PCR primers that resulted in amplification of a sequence in all 15 *C. posadasii* isolates but not isolates of *C. immitis* (Figure 2). 

## 4. Discussion

The isolates employed in this study came from individuals across New Mexico, with several isolates coming from the northwestern part of the State (San Juan County, NM, USA; Table 1). This is a surprising result given that species of *Coccidioides* are expected to occur primarily in the southern portions of New Mexico, where the environment is similar to the Sonoran Desert regions of Arizona and California. Although 14 of the 15 patients who were infected with *C. posadasii* were residents of New Mexico, two reported recent travel to Arizona (Table 1). New Mexico sees far fewer cases of coccidioidomycosis than its neighbor Arizona (approximately 47 cases/yr compared to 9680 cases/yr from 2008–2014) [23]. There are three non-mutually-exclusive possible reasons for this: (1) physicians in New Mexico do not have a thorough understanding of the disease for diagnosis and treatment, (2) *Coccidioides* is most common in less populated areas of the state, and/or (3) the low human population density across most portions of the state means that there are fewer targets for infection and less human-caused soil disturbance on a wide scale (a known factor in generating the airborne spores that cause infections). From a Knowledge, Attitudes, and Practices Survey of New Mexican physicians in 2010, 72% were not confident in their ability to diagnose and 70% were not confident in their ability to treat coccidioidomycosis. There is a clear need for an increased understanding of disease ecology, including the environmental conditions related both to where the fungus grows and factors that aid in dispersion to better inform our healthcare professionals of the distribution in New Mexico. An increased awareness will help avoid complications from delayed diagnosis and inappropriate treatment that can result in severe disease progression and even death. This point is driven home by a news report of several recent severe cases of human coccidioidomycosis in Southern New Mexico, all of which resulted in delayed diagnosis that prevented timely treatment [24].

Native Americans and African Americans represent only 11% and 2.5% of the population of New Mexico, respectively [25], so it was surprising to see five of eight infections for which patient ethnicity was known occurred in Native Americans, and two of the eight occurred in African Americans (Table 1). The increased risk for coccidioidomycosis among African Americans is well known [26]. The risk among Native Americans is not documented in the literature. Although one study [27] found Southwest Native Americans in regions of high *Coccidoides* endemism (Lower Sonoran desert) to have high rates of positive response to coccidioidin skin tests, that study did not find that rates were higher than for non-Native Americans in the same region. A separate study found a possible correlation between diabetes and coccidioidomycosis-associated death among Native Americans [28]. We acknowledge that any or all of multiple factors could contribute to increased risk of coccidioidomycosis. These include living in remote rural areas with high airborne dust loads, working in agriculture or construction, as well as comorbidities such as diabetes and other health conditions. Nonetheless, our results suggest a need for health professionals in New Mexico and the Four Corners region to be aware of potential at-risk groups, as well as a need for a better understanding of locations of *Coccidioides* endemism.

Coccidioidomycosis incidence is on the rise in highly endemic areas such as Arizona and California and in more sparsely populated reporting regions including New Mexico, Nevada, and Utah [11]. One reason for this increase may be that clinicians are becoming more aware of the disease. Other hypotheses include changes in testing practices, increased travel or relocation to endemic areas, and/or growth of the “at-risk” immunosuppressed population (although coccidioidomycosis can infect healthy individuals). Climatic factors, such as temperature and moisture, in addition to increases in human activities such as construction that produce dust from soil disturbance, can result in increased spore dispersal [29]. Our genetic analysis of isolates collected from patients diagnosed with coccidioidomycosis in New Mexico provides a foundation for future exploration of distribution, incidence, and susceptibility of patients in New Mexico and the American Southwest Four Corners region.

Because *C. immitis* has been considered to be the “California species” and the vast majority of *C. immitis* infections occur in California, the presence of *C. immitis* among our isolates was unexpected. The reported range for *C. immitis* does, however, include locations in Washington state and Northeastern Utah [13,30]. Acknowledging that the *C. immitis* isolates we examined could reflect infections acquired as a result of travel outside the Four-Corners region, it is entirely possible that the range of *C. immitis* includes Southern Utah and/or Northern New Mexico. Related to the question of species distributions, we note that although hybridization between *C. immitis* and *C. posadasii* has been reported [31,32], the fact that all four nuclear genes and the mitochondrial region examined for our isolates agreed in terms of species separations would suggest that any introgression of genes across species would be minimal for the isolates we examined.

Our observation that *C. immitis* was present among isolates obtained from patients in New Mexico argues that it is important for health professionals and researchers to have rapid methods to distinguish between the two species. This is true in part because, while differences in distribution of the two species have become increasingly clear in the past decade, the ecological niche difference between the species is unknown [33,34]. Although physicians do not currently rely on speciation for diagnosis and treatment, this may well change in the future with the increasing discovery of genetic and phenotypic differences between the species. For example, *C. immitis* has a tendency to grow faster than *C. posadasii* on high-salt media [6], which suggests there may be other growth differences in physiology that affect virulence and ecology. Rapid methods to distinguish between the two *Coccidioides* species such as the PCR-based method we employed here with mtDNA, along with similar methods that can be used to detect species of *Coccidioides* in environmental DNA samples, should prove valuable in future studies. Given the reported hybridization between *C. immitis* and *C. posadasii* cited above [31,32], we acknowledge that species assignments made based on a single gene region should be viewed as tentative.

## Figures and Tables

**Table 1:** Isolate and patient information.

Isolate	Isolate ID ^a^	County or State of Residency	Source Type of Infection	Age	Sex	Race/Ethnicity	Significant Past Medical History	Occupation	Travel History
***C. immitis* Isolates**									
NM3006	*Ci*	San Juan, NM	Bone Vertebral osteomyelitis	60	M	American Indian	Yes	Railroad employee	None
NM9443 ^b^	*Ci*	Utah	Ear	51	M	Unk ^c^	Unk	Unk	Unk
NM9737 ^b^	*Ci*	Utah	Ear	51	M	Unk	Unk	Unk	Unk
***C. posadasii* Isolates**									
NM0369	*Cp*	Bernalillo, NM	Bronchial wash Pulmonary infection	66	M	Unk	Yes	Unk	None
NM4233	*Cp*	Bernalillo, NM	Tissue Pulmonary infection	48	M	Unk	Yes	Rug merchant	Tucson, AZ, 3 wks prior
NM9861	*Cp*	Bernalillo, NM	Tissue Pulmonary infection	30	F	Unk	Yes	Unk	None
NM3894	*Cp*	Bernalillo, NM	Fluid Knee infection	77	M	Unk	No	None	None
NM0317	*Cp*	Chaves, NM	Ear Pulmonary infection and facial lesion	62	M	African American	Yes	Retired. Prior military in CA	None
NM4297	*Cp*	Eddy, NM	Sputum Pulmonary infection	63	M	White	Yes	Mining engineer	Travel to Kansas
NM0071	*Cp*	Lea, NM	Nasal sputum Pulmonary infection	28	M	African American	Yes	Incarcerated patient	N/A
NM3957	*Cp*	McKinley, NM	Pleural fusion Pulmonary infection	42	M	Unk	Unk	Unk	Unk
NM9837	*Cp*	McKinley, NM	Nasal sputum Pulmonary infection	51	M	American Indian	Yes	Welder	Recent work Phoenix, AZ
NM5945	*Cp*	McKinley, NM	Tissue Pulmonary infection	48	F	American Indian	Yes	Unk	None
NM4708	*Cp*	San Juan, NM	Bronchial wash Pulmonary infection	40	F	American Indian	Yes	Unk	None
NM0459	*Cp*	Socorro, NM	Tissue Pulmonary infection	50	F	Unk	Unk	Unk	None
NM7898	*Cp*	Torrance, NM	Bronchial lavage Pulmonary infection	48	M	Unk	Unk	Incarcerated patient	None
NM8725	*Cp*	New Mexico	Fluid/Unk	Unk	Unk	Unk	Unk	Unk	Unk
NM8945	*Cp*	Arizona	Bone Pulmonary infection in childhood; osteomyelitis	62	M	American Indian	Yes	Unk	None

^a^*Ci* = *C. immitis*, *Cp* = *C. posadasii*; ^b^ NM9443 and NM9737 were from the same patient; ^c^ Unk = unknown.

**Table 2:** Primers used in gene amplification.

DNA Region	Direction	Sequence	Annealing Temp.
ITS	Forward	5′ CTTGGTCATTTAGAGGAAGTAA 3′	50 °C
	Reverse	5′ TCCTCCGCTTATTGATATGC 3′	
Serine Proteinase	Forward	5′ ATAGAGACCACGCAGAAGGC 3′	55 °C
	Reverse	5′ AGCTGTCACGGATGGTATCG 3′	
MCM7	Forward	5′ TGGTTATAGCGCGATTCTCC 3′	50 °C
	Reverse	5′ CGAGTCGTTATACCTCGAACG 3′	
RPB1	Forward	5′ CCGCGTCATTATTTAAGCATC 3′	55 °C
	Reverse	5′ AGCGTATTCACCAACTTCTC 3′	
*C. posadasii* Intron ^a^	Forward (P2F)	5′ TCAAATCATGTGTAATATGTGG 3′	50 °C
	Reverse (P2R)	5′ GTTGACCATAAAGAAAAGTTGG 3′	
cox1 exon ^b^	Forward (P1F)	5′ ATAAAATAAACTACGATTTGCG 3′	50 °C
	Reverse (P1R)	5′ GATTGCATGAGCTGTAATAATAC 3′	

^a^ This primer pair amplifies an intron sequence from *C. posadasii* but not C. *immitis* (see Results). ^b^ This primer pair amplifies a region in both *C. posadasii* and *C. immitis* that includes a portion of the first cox1 exon along with an upstream intergenic region (see Section 3).

**Table 3:** GenBank accession numbers for sequences employed in phylogenetic analysis.

Isolate	ITS	Serine Proteinase	MCM7	RPB1
**Sequences from New Mexico and Four Corners Region *Coccidioides* Isolates (This Study)**				
NM3006	MH725248	MH748764	MH748742	MH748724
NM9443	MH725258	MH748774	MH748757	MH748739
NM9737	MH725259	MH748775	MH748758	MH748740
NM0071	MH725244	MH748760	MH748749	MH748731
NM0317	MH725245	MH748761	MH748748	MH748730
NM0369	MH725246	MH748762	MH748759	MH748741
NM0459	MH725247	MH748763	MH748746	MH748728
NM3894	MH725249	MH748765	MH748754	MH748736
NM3957	MH725250	MH748766	MH748756	MH748738
NM4233	MH725251	MH748767	MH748743	MH748725
NM4297	MH725252	MH748768	MH748752	MH748734
NM4708	MH725253	MH748769	MH748744	MH748726
NM5945	MH725254	MH748770	MH748751	MH748733
NM7898	MH725255	MH748771	MH748747	MH748729
NM8725	MH725256	MH748772	MH748755	MH748737
NM8945	MH725257	MH748773	MH748750	MH748732
NM9837	MH725260	MH748776	MH748745	MH748727
NM9861	MH725261	MH748777	MH748753	MH748735
**Sequences from Existing GenBank *Coccidioides* Entries**				
RMSCC 2394	AATX01000513.1	AATX01000326.1	AATX01000203.1	AATX01000264.1
	224–846	35438–36110	43422–44141	433820–434247
H538.4	AASO01000085.1	AASO01002025.1	AASO01002210.1	AASO01003054.1
	10339–10766	35115–35787	4641–5360	10945–11568
RS	AAEC03000009.1	AAEC03000008.1	AAEC03000005.1	AAEC03000010.1
	4725–5351	2537048–2537720	3687326–3688045	828825–829252
CPA 0020	ABIV01003320.1	ABIV01000896.1	ABIV01000569.1	ABIV01001762.1
	16973–17598	3088–3760	311–1030	5431–5860
CPA 0001	ABFO01003353.1	ABFO01000797.1	ABFO01001238.1	ABFO01003988.1
			1–346 ^a^	
	2878–3503	665–1337	ABFO01001237.1	6129–6558
			12376–12757 ^a^	
RMSCC 3700	ABFN01001891.1	ABFN01000336.1	ABFN01000290.1	ABFN01001137.1
	3121–3746	1–638	5242–5961	412–841
RMSCC 2133	ABFM01000924.1	ABFM01000717.1	ABFM01000464.1	ABFM01000297.1
	22927–23552	3481–4153	1904–1262	6563–6992
RMSCC 3488	ABBB01000249.1	ABBB01000240.1	ABBB01000156.1	ABBB01000255.1
	33179–33804	371029–371701	145000–145719	527482–527911
Silveira	KM588216.1	ABAI02000152.1	ABAI02000361.1	ABAI02000102.1
		34354–35026	12054–12773	54472–54901
**Sequences from Existing GenBank *Aspergillus steynii* Entries**				
IBT 23096	MSFO01000033.1	MSFO01000001.1	MSFO01000005.1	MSFO01000005.1
	3264–2712	3181742–3182796	1831768–1834777	2491035–2491482

^a^ The complete CPA0001 MCM7 gene is split across two contig accessions.

## Supplementary Materials

Supplementary materials can be found at https://www.mdpi.com/2309-608X/5/3/74/s1. Figure S1. Phylogenetic trees for individual gene regions: Serine protease, MCM7, RPB1, and ITS.
