# Supplementary material for: First Analysis of Human Coccidioides Isolates from New Mexico and the Southwest Four Corners Region: Implications for the Distributions of C. posadasii and C. immitis and Human Groups at Risk
Source: J Fungi (Basel). 2019 Aug 10;5(3):74. doi: 10.3390/jof5030074 (PMC6787932; doi:10.3390/jof5030074)
Supplement: Supplementary file 1 [file jof-05-00074-s001.pdf]

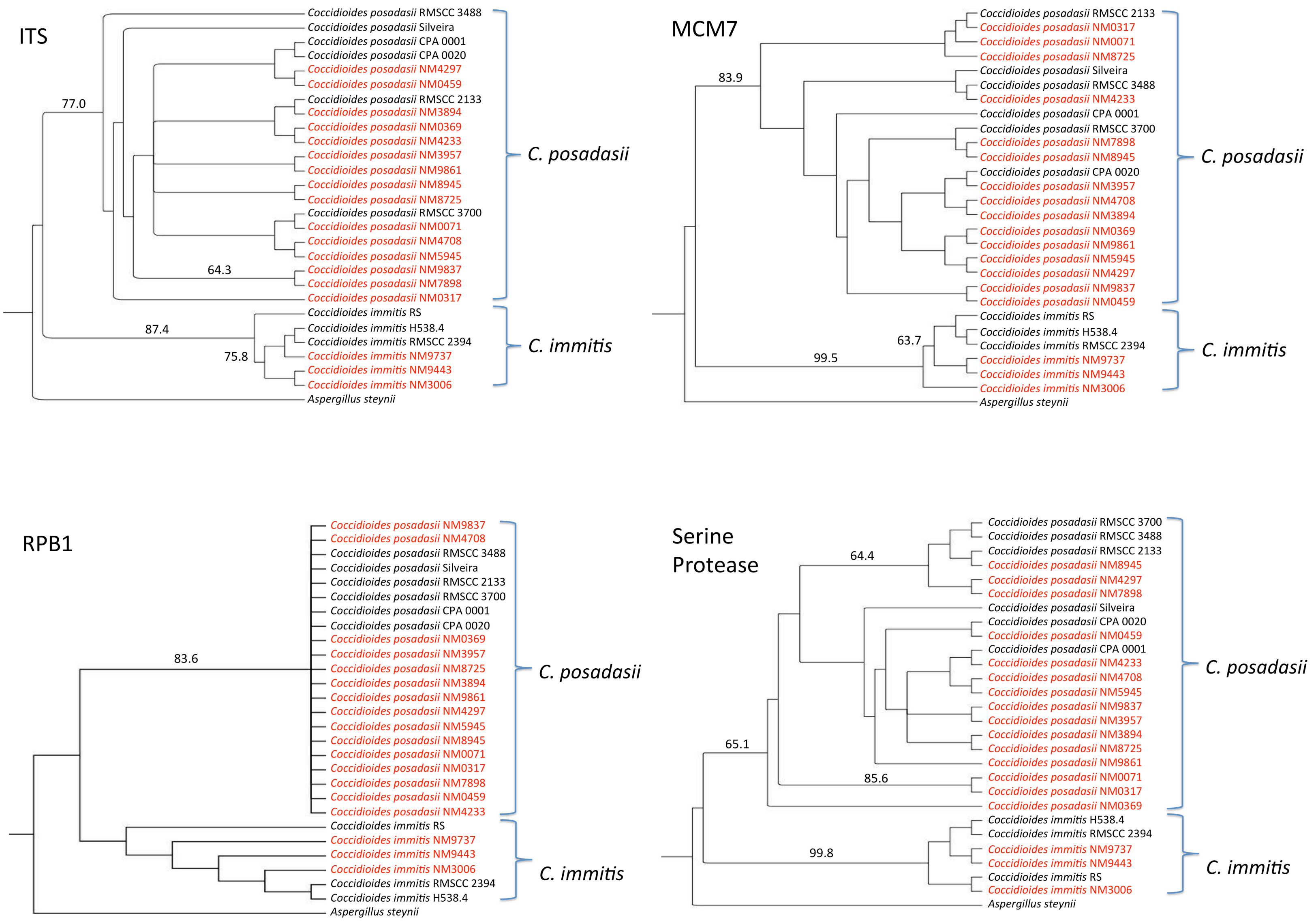

Figure S1. Phylogenetic trees (DNAMLK) for the four individual gene regions examined for *Coccidioides* isolates from New Mexico and the Four Corners region (shown in red). The trees were derived from alignments of partial sequences from four gene regions: ITS, MCM7, RPB1, and serine protease. Procedures for obtaining alignments and tree building were the same as for the tree presented in Figure 1. Bootstrap values are shown for branches with greater than 60% support (1000 replicates). The tree was rooted with *Aspergillus steynii*. Note that all trees show separate groupings for *C. immitis* and *C. posadasii* with good bootstrap support.
